# Transcriptomic Signatures of Single-Suture Craniosynostosis Phenotypes

**DOI:** 10.3390/ijms24065353

**Published:** 2023-03-10

**Authors:** Samantha Lapehn, Jonas A. Gustafson, Andrew E. Timms, Michael L. Cunningham, Alison G. Paquette

**Affiliations:** 1 Center for Developmental Biology & Regenerative Medicine, Seattle Children’s Research Institute, Seattle, WA 98101, USA; samantha.lapehnyoung@seattlechildrens.org (S.L.);; 2 Department of Pediatrics, University of Washington, Seattle, WA 98195, USA

**Keywords:** craniosynostosis, RNA sequencing, homeobox, transcriptome

## Abstract

Craniosynostosis is a birth defect where calvarial sutures close prematurely, as part of a genetic syndrome or independently, with unknown cause. This study aimed to identify differences in gene expression in primary calvarial cell lines derived from patients with four phenotypes of single-suture craniosynostosis, compared to controls. Calvarial bone samples (N = 388 cases/85 controls) were collected from clinical sites during reconstructive skull surgery. Primary cell lines were then derived from the tissue and used for RNA sequencing. Linear models were fit to estimate covariate adjusted associations between gene expression and four phenotypes of single-suture craniosynostosis (lambdoid, metopic, sagittal, and coronal), compared to controls. Sex-stratified analysis was also performed for each phenotype. Differentially expressed genes (DEGs) included 72 genes associated with coronal, 90 genes associated with sagittal, 103 genes associated with metopic, and 33 genes associated with lambdoid craniosynostosis. The sex-stratified analysis revealed more DEGs in males (98) than females (4). There were 16 DEGs that were homeobox (HOX) genes. Three TFs (SUZ12, EZH2, AR) significantly regulated expression of DEGs in one or more phenotypes. Pathway analysis identified four KEGG pathways associated with at least one phenotype of craniosynostosis. Together, this work suggests unique molecular mechanisms related to craniosynostosis phenotype and fetal sex.

## 1. Introduction

Craniosynostosis is the premature fusion of one or more of the calvarial sutures, that occurs in syndromic and non-syndromic forms in approximately 1 in 2100–2500 live births [1,2,3]. The syndromic craniosynostoses are hereditary conditions, defined as having craniosynostosis associated with reproducible features involving non-craniosynostosis phenotypes, such as limb or facial malformations. Craniosynostosis has been associated with over 150 different syndromes [4,5]; however, isolated single-suture fusions account for approximately 85% of all patients diagnosed with craniosynostosis [6]. Single suture craniosynostosis (SSC) is defined as premature fusion of one of the four major sutures of the calvaria (metopic, sagittal, coronal, or lambdoid). Single-suture sagittal and metopic craniosynostosis have a nearly four-fold increased incidence among males, whereas in single-suture coronal cases there is a small increased incidence among females [7]. Single-suture craniosynostosis (SSC) follows Mendelian patterns of inheritance in some families, with approximately 6–8% of patients having a positive family history that is consistent with autosomal dominant transmission [8,9]. Cases with family recurrence usually involve the same suture, but there have been large pedigrees with coronal or sagittal synostosis that exhibit significant intrafamilial variability [7,8]. Beyond strictly genetic causes, abnormal in utero mechanical forces [10,11,12,13,14] and in utero exposure to valproic acid [9], are risk factors for the development of craniosynostosis. Craniosynostosis can be accompanied by a variety of medical conditions, most commonly including hydrocephalus, increased intracranial pressure, intellectual disabilities, and visual or hearing deficits [15]. Therefore, craniosynostosis is one of the most clinically significant craniofacial disorders due to its prevalence, associated morbidity, complex surgical needs, and related burden on families and healthcare.

Although the molecular pathogenesis for the majority of rare syndromic forms of craniosynostosis has been identified, the causes of the more common isolated single-suture forms remain effectively unknown. RNA sequencing technology has the capability to uncover novel genes and pathways related to single-suture craniosynostosis. To date there has not been a transcriptome-wide evaluation of gene expression changes in single-suture phenotype of craniosynostosis. Previous investigations of transcriptomic changes in craniosynostosis have largely utilized microarray or RT-qPCR-based approaches, which are biased toward previously identified gene associations. Conversely, RNA sequencing analysis allows for an unbiased evaluation of the whole transcriptome, with the potential to identify unknown gene associations and patterns of expression across gene family groups and biological pathways.

This study will evaluate the craniosynostosis transcriptome across four single-suture phenotypes (coronal, metopic, sagittal, lambdoid) in a cohort of patients (N = 388 cases, 85 controls) recruited through four study sites in the United States between 2002 and 2016, with RNA sequencing performed on primary calvarial cell lines derived from these patients. The aim of this paper is to identify transcriptomic signatures (including sex-specific associations) of single-suture craniosynostosis phenotypes, and we hypothesize that each phenotype will be associated with a unique subset of genes and pathways, with differences related to patient sex.

## 2. Results

### 2.1. Study Participant Characteristics

RNA was sequenced from cultured primary calvarial cells derived from 85 controls and 388 craniosynostosis cases, that included 80 coronal, 196 sagittal, 92 metopic, and 20 lambdoid phenotypes (Table 1), as well as 85 control samples without craniosynostosis. As expected, the coronal phenotype was over-represented in females, and the metopic and sagittal phenotypes were over-represented in males (chi-squared test *p*-value < 0.05, Table 1). There were no statistically significant differences across craniosynostosis phenotypes for proband age, at the time of sample collection. However, the age of control patients at time of collection was significantly different from all four craniosynostosis groups (*p* < 0.001 for each phenotype vs. control comparisons, ANOVA with Tukey’s post hoc test) (Table 2). There was no statistically significant difference in cell culture time between controls and craniosynostosis phenotypes (ANOVA *p* > 0.05) (Table 2). 

### 2.2. DEGs and Pathway Analysis by Phenotype

**Figure 1:**
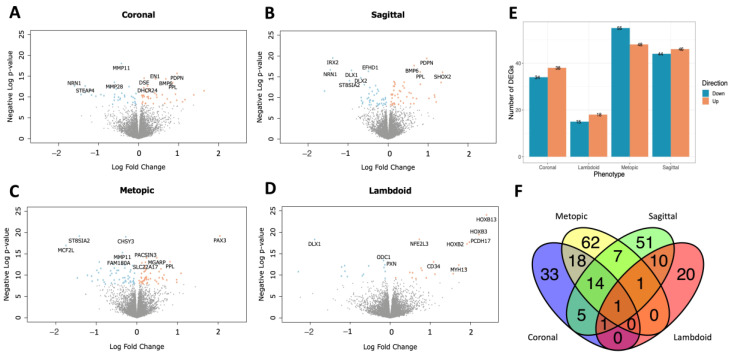
Differentially expressed genes (DEGs) (FDR < 0.05) for the primary model. DEGs are represented as blue (decreased) or orange (increased) points. The top 10 DEGs, based on log fold change, are labeled by gene ID. (**A**) Coronal phenotype, (**B**) sagittal phenotype, (**C**) metopic phenotype, (**D**) lambdoid phenotype, (**E**) number and direction of DEGs for each phenotype, (**F**) overlap of DEGs across phenotypes.

In our primary model, we identified 72 DEGs whose expression was associated with the coronal phenotype of craniosynostosis, 33 DEGs associated with lambdoid craniosynostosis, 103 DEGs associated with metopic craniosynostosis, and 90 DEGs associated with sagittal craniosynostosis, compared to controls without craniosynostosis. The full results are presented in Supplemental Table S1. Generally, we observed an even distribution of DEGs that were increased and decreased in craniosynostosis phenotypes vs. controls (Figure 1). The top three increased DEGs associated with each phenotype were, *PAX3*, *GATA3*, and *OLR1* (coronal); *PAX3*, *TFAP2A*, and *ALX1* (metopic); *SHOX2*, *HOXB13*, and *CPAMD8* (sagittal); and *HOXB13*, *HOXB3*, and *PCDH17* (lambdoid). The top three decreased DEGs associated with each phenotype were, *NRN1*, *MCF2L*, and *STEAP4* (coronal); *MCF2L*, *SMOC2*, and *ST8SIA2* (metopic); *CCKAR*, *NRN1*, and *IRX2* (sagittal); and *TRH*, *DLX1*, and *XIRP1* (lambdoid). There was a single gene, *EFHD1*, that was significantly decreased across all four phenotypes of craniosynostosis compared to controls (Figure 1F). The lambdoid and metopic phenotypes had the highest percentage of unique DEGs (lambdoid = 20/33 unique DEGs, metopic = 62/103 unique DEGs) that were not significantly associated with other craniosynostosis phenotypes. There were 51 genes (57% of sagittal DEGs) uniquely associated with sagittal craniosynostosis, and 33 genes (46% of coronal DEGs) uniquely associated with coronal craniosynostosis (Figure 1F).

Using rotational gene set testing, we identified three significant pathways (FDR < 0.05) whose expression was associated with the coronal phenotypes (Table 3). All three pathways exhibited increased expression compared to controls, and are categorized as metabolism pathways by KEGG.

### 2.3. Sex-Stratified DEGs and Pathways by Phenotype

In our models constructed in male infants alone (N = 318), we identified 36 DEGs associated with sagittal craniosynostosis, 68 DEGs associated with metopic craniosynostosis, 6 DEGs associated with lambdoid craniosynostosis, and 9 DEGs associated with coronal craniosynostosis (Supplemental Figure S1, full results in Supplemental Table S1). None of the DEGs were significant in all four phenotypes in males. We identified one significant pathway (FDR < 0.05)—“Growth hormone synthesis, secretion, and action”—with increased expression associated with metopic craniosynostosis in males (Table 3). There were no other pathways significantly increased or decreased for phenotypes in the male-stratified models, compared to controls.

In our models constructed in female infants alone (N = 155), we identified fewer DEGs (FDR < 0.05) for each phenotype than our male-stratified analysis, including two DEGs associated with coronal (*NR3C2*, *MMP11*), one DEG associated with sagittal (*CD74*), one DEG associated with metopic *(NR3C2*), and one DEG associated with lambdoid craniosynostosis (*RBM20*) (Supplemental Figure S2, Supplemental Table S1). All DEGs showed decreased expression in the female craniosynostosis phenotypes, compared to female controls. One gene, *NR3C2*, was significantly decreased in two phenotypes (coronal and metopic). There were no significant pathways identified in the female model. 

**Figure 2:**
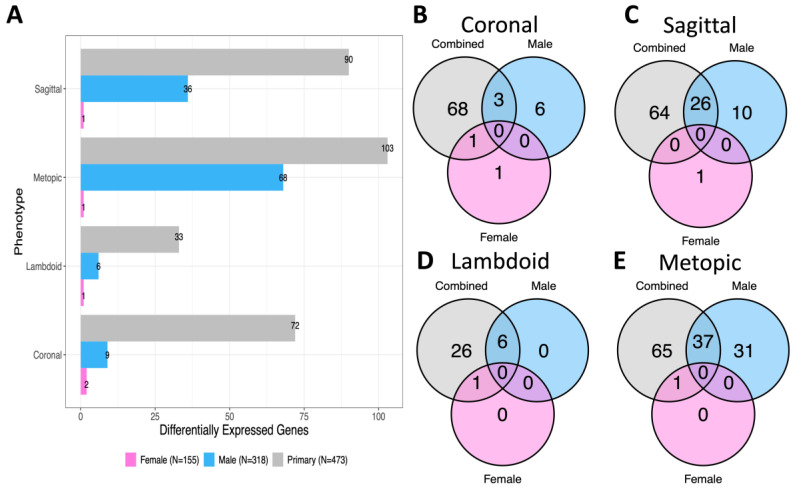
Comparison of differentially expressed genes (DEGs) for each phenotype model. (**A**) Comparison of DEG number across combined-sex, male-stratified, and female-stratified phenotype models. (**B**) Overlap of DEGs across models in coronal phenotype. (**C**) Overlap of DEGs across models in sagittal phenotype. (**D**) Overlap of DEGs across models in lambdoid phenotype. (**E**) Overlap of DEGs across models in metopic phenotype.

Across all phenotypes, more genes were significantly associated with craniosynostosis in the primary model (N = 473) than either the male (N = 318) or female (N = 155) sex-stratified models (Figure 2A). We did not identify any significant genes within a phenotype that were shared across our independent models for males, females, and the combined-sex analysis (Figure 2B–E). In all four craniosynostosis phenotypes, there were a subset of DEGs that were significant in both the male-stratified and primary models, while there was only overlap between the female-stratified and primary models for three phenotypes (coronal, lambdoid, metopic) (Figure 2B–E).

**Figure 3:**
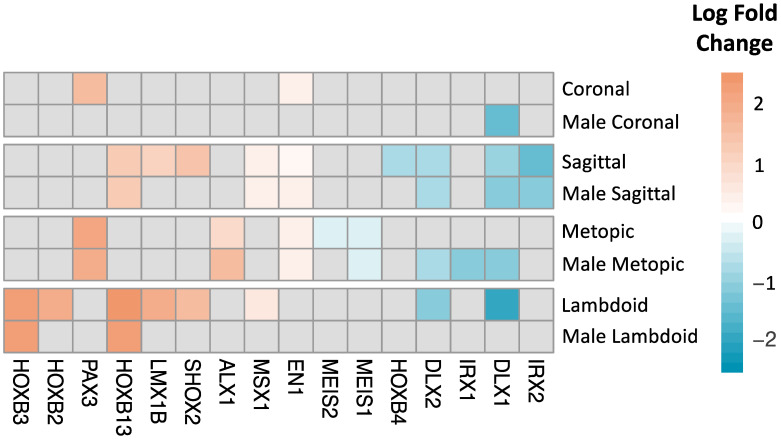
Homeobox DEGs for combined-sex and male-stratified phenotype models, shaded by log fold change. No homeobox genes were identified as DEGs in the female-stratified model.

Several homeobox genes were among the most differentially expressed genes (by log fold change) in the male and combined models, prompting a deeper characterization of homeobox genes. In total, there were sixteen DEGs associated with craniosynostosis phenotypes that were members of the homeobox gene family [16], including two DEGs associated with coronal craniosynostosis, eight DEGs associated with lambdoid craniosynostosis, five DEGs associated with metopic craniosynostosis, and nine DEGs associated with sagittal craniosynostosis (Figure 3). All 16 homeobox genes showed directional concordance in log fold changes for each phenotype to control comparison. There were seven homeobox genes exhibiting decreased expression and nine homeobox genes exhibiting increased expression. In the primary model, the homeobox genes with the greatest overlap across phenotypes were *DLX1*, *DLX2* (decreased for sagittal and lambdoid), *HOXB13* (increased for sagittal and lambdoid), and *EN1* (increased for metopic, sagittal, and coronal). In the male-stratified model, *DLX1* (decreased) was the only homeobox gene significantly associated with the three phenotypes (coronal, metopic, sagittal). There were a few genes that were only significantly associated with a single craniosynostosis phenotype, including *HOXB4* (sagittal), *IRX2* (sagittal and male sagittal), *MESI1*, *MEIS2* (metopic), *IRX1* (male metopic), *ALX1* (metopic and male metopic), *HOXB2* (lambdoid), and *HOXB3* (lambdoid and male lambdoid).

### 2.4. Transcription Factor Enrichment Analysis

**Figure 4:**
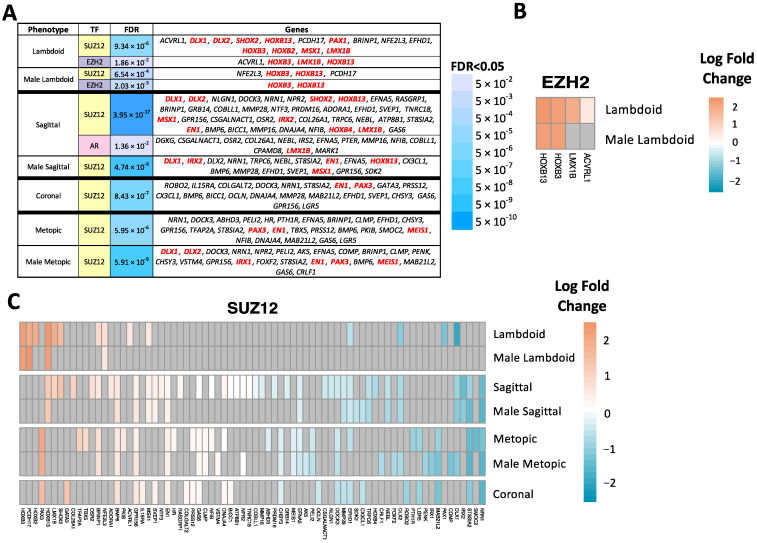
Transcription factor enrichment analysis with Enrichr. (**A**) Significant transcription factors (TFs) (FDR < 0.05) for the combined-sex and male-stratified phenotype models identified through enrichment analysis with Enrichr. Red genes are homeobox genes. (**B**) Differentially expressed downstream genes of EZH2, shaded by log fold change (**C**) Differentially expressed downstream genes of SUZ12, shaded by log fold change.

Three unique transcription factors emerged as potential regulators of DEGs, whose expression was associated with different craniosynostosis phenotypes in our primary and male-stratified models. These TFs were determined based on over-representation of TF binding sites in regulatory regions of craniosynostosis DEGs, measured through Enrichr (Figure 4A, FDR < 0.05). Two TFs, SUZ12 and EZH2, were significantly enriched as regulators of DEGs from more than one model or phenotype. These TFs are both members of the polycomb repressive complex (PRC) [17]. SUZ12 was significantly enriched for DEGs associated with lambdoid, sagittal, metopic, and coronal craniosynostosis from the primary model, and sagittal, metopic, and lambdoid craniosynostosis in the male sex-stratified model. All downstream DEGs of SUZ12 were directionally concordant across models and phenotypes, with 37 DEGs exhibiting increased expression and 37 DEGs exhibiting decreased expression, in craniosynostosis phenotypes compared to controls (Figure 4C). EZH2 was significantly enriched for DEGs associated with lambdoid craniosynostosis in the primary model and male sex-stratified model. The downstream genes regulated by EZH2 were all upregulated (Figure 4B). Three of the DEGs downstream of EZH2 (*HOXB3*, *HOXB13*, *LMX1B*) are members of the HOX family.

## 3. Discussion

This paper aimed to identify transcriptomic signatures of four single-suture phenotypes of craniosynostosis. This was accomplished through RNA sequencing of 473 patient- and control-derived primary calvarial cell lines, followed by differential gene expression and pathway analysis. The key findings of this study were that, (1) each craniosynostosis phenotype had at least 20 unique differentially expressed genes (DEGs), (2) more DEGs were identified in the male-stratified analysis compared to the female-stratified analysis, and (3) that homeobox genes were prevalent as DEGs across the primary and male-stratified models. Overall, these findings highlight the importance of assessing gene expression by phenotype and sex. Results also identified novel genes and pathways whose expression is altered in these single-suture craniosynostosis phenotypes.

In the primary model, DEGs were identified for all four phenotypes with an approximately equal number of increased and decreased genes. Lambdoid craniosynostosis has the lowest number of DEGs (33 DEGs), while metopic craniosynostosis has the highest number of DEGs (103 DEGs). Several of the DEGs with the largest changes in expression have previously been associated with craniosynostosis or osteoblast differentiation. *SMOC2* was among the most decreased genes in the metopic phenotype, compared to controls, and was also significantly decreased in the male-stratified metopic analysis. In canines with brachycephaly, *SMOC2* is downregulated, due to alternative splicing caused by a LINE-1 insertion, which may indicate a role for *SMOC2* in bicoronal (or brachycephalic) pediatric craniosynostosis [18]. Conversely, *TFAP2A* was among the most increased DEGs in metopic craniosynostosis patients, compared to controls, and although it does not have a known association with craniosynostosis, its family of genes (TFAP) has been associated with other craniofacial deficiencies in tissues from a neural crest lineage [19], including branchio-oculo-facial syndrome [20], Char syndrome [21], and neural tube and skeletal defects [22]. Additionally, *TFAP2A* expression in mesenchymal cells derived from the sagittal suture, has been measured as 1.97 times higher than in mesenchymal cells from the metopic suture [19]. *GATA3* was among the most increased DEGs associated with coronal craniosynostosis, compared to controls, and has previously been shown to have roles in osteoblast survival [23] and the healing of bone fractures [24], which could explain its increased expression seen in the coronal craniosynostosis phenotype. 

There are known differences in incidence by patient sex across single-suture craniosynostosis phenotypes [25,26,27]. We identified distinct differences in gene expression changes by patient sex, with male patients having vastly more DEGs than female patients for each craniosynostosis phenotype. Female models only identified 1–2 DEGs per phenotype, while male model DEGs ranged from 6 to 68. For every phenotype except lambdoid, there were also DEGs that were uniquely significant in the sex-stratified models compared to the primary model, for the same phenotype. The etiology of the male preponderance of craniosynostosis is not fully understood [26], but is an active area of research. A previous paper studying a subset of the same cohort of patients presented herein, evaluated differences in alkaline phosphatase (ALP) activity by phenotype and sex [25]. The sagittal and metopic phenotypes in males, and the sagittal phenotype in females, was associated with increased Alp activity, and each of these phenotypes had unique gene sets, identified by microarray, that were correlated with Alp activity status [25]. The *ALPL* gene, that encodes alkaline phosphatase, was significantly increased in metopic craniosynostosis in our primary and male-stratified models (Supplemental Table S1). Multiple genetic variants of interest within *ALPL* have previously been identified, and are associated with occasional (<50%) occurrence of single-suture craniosynostosis [28]. To our knowledge, the present study represents the first RNA sequencing evaluation of craniosynostosis phenotypes, in primary calvarial cell lines, to evaluate global gene differences by sex, so further research is needed to fully evaluate the differences in expression. 

Several of the genes identified through the sex-stratified analysis were also related to craniosynostosis or osteoblast differentiation. *BMP6* was one of the most increased genes in the male sagittal phenotype and was also significant in the male metopic phenotype, and the coronal, sagittal, and metopic phenotypes in the primary model. BMP proteins comprise a gene family of over 22 members, that are components of the TGF-beta signaling pathway, where they are involved in the differentiation of mesenchymal stem cells to osteoblasts [29]. In a case study of a prenatal diagnosis of de novo pure trisomy 6p (6p22.3 → p25.3) affected with craniosynostosis and microcephaly, the patient had a 20.88 Mb dosage increase in the genomic region containing the gene *BMP6*, which led to overexpression of this gene [30]. Recent studies have revealed that damaging de novo variants of genes within the BMP signaling pathway are associated with lambdoid craniosynostosis in human [31], and that augmented BMP signaling in mice neural crest cells is associated with premature fusion of intersphenoid synchondroses, resulting in craniofacial anomalies including craniosynostosis [32]. *CLDN11* was significantly increased, compared to controls, in coronal craniosynostosis in males, and was one of the top three increased genes in our male-stratified coronal craniosynostosis model. Although this gene has not previously been associated with craniosynostosis, *CLDN11* is robustly expressed during osteoblast differentiation. Mouse osteoblasts that were treated with ascorbic acid for up to 24 days, to induce differentiation, exhibited a 60-fold increase in *Cldn11* expression between days 0 and 8, which then decreased toward the later stages of differentiation [33]. These results were specific to osteoblast cells, as osteoclast cells exhibited decreased *CLDN11* expression, indicating that *CLDN11* expression is dependent on cell type and stage of differentiation [33]. Treatment with a recombinant *CLDN11* protein has been demonstrated to prevent bone density impairment that is caused by LPS injection [34]. 

*MMP11* exhibited decreased expression in female coronal craniosynostosis compared to controls and was also decreased in the primary models for the coronal, metopic, and sagittal phenotypes, and male models for metopic and sagittal phenotypes. The MMP gene family degrades extracellular matrix proteins, allowing for cell migration, and also has roles in cell attachment, proliferation, differentiation, and apoptosis [35]. *MMP11* is part of the stromelysin sub-family of MMPs and has been identified, through histologic staining of the growth plate, in osteoblasts, osteocytes, and chrondrocytes in the proliferative and hypertrophic zones [35].

Across both the primary and sex-stratified analyses, many of the DEGs for each phenotype were members of the homeobox gene family, which are a class of genes that contain a homeodomain DNA sequence [16]. Many of the proteins encoded by these genes act as transcription factors that are important for embryonic development [16]. While homeobox genes as a class have not been broadly associated with craniosynostosis, there is evidence for the involvement of a few homeobox genes in craniosynostosis etiology, including several identified in our transcriptome analysis.

*MSX2* and *MSX1* are homeobox genes involved in craniofacial development [36], that have previously been associated with craniosynostosis. An *MSX2* gain of function mutation is associated with Boston-type craniosynostosis, which most commonly affects lambdoid and coronal sutures [37]. *MSX2* overexpression in mice is associated with sagittal suture fusion [38]. Although *MSX2* was not identified as a DEG in this analysis, *MSX1*, whose potential role in craniosynostosis is not well-defined, was increased in lambdoid and sagittal craniosynostosis in the primary model and sagittal craniosynostosis in the male-stratified model. MSX1+ skeletal stem cells are also able contribute to bone regeneration and ossification in response to calvarial defect injuries in rats, when treated with an optimized neurotrophic supplement [39].

*ALX4* is expressed in the mesenchyme of developing bone and has several connections to calvarial ossification and craniosynostosis. Genetic variants of *ALX4* have been linked to craniosynostosis across multiple studies [40,41,42]. Mutations in *ALX4* have also been associated with other craniofacial defects including enlarged parietal foramina, which is an ossification defect within the parietal bone, highlighting the role of *ALX4* in calvarial ossification during development [43]. Though *ALX4* was not differentially expressed between cases and controls in our study, *ALX1* was significantly increased in metopic craniosynostosis in our primary and male-stratified models. *ALX1* has not previously been associated with craniosynostosis, however, it is involved in osteogenesis and regulates a lncRNA transcript that is involved in bone marrow mesenchymal cell osteogenesis [44].

Pathway analysis was performed, using FRY, on KEGG gene sets, to extend the biological significance of the findings to gene networks. The three pathways associated with coronal craniosynostosis in the primary model are all related to metabolic processes, but have not previously been associated with craniosynostosis, while the single pathway associated with metopic craniosynostosis in males was growth hormone, synthesis, secretion, and action. Although growth hormone is not directly implicated in craniosynostosis etiology, it is known to play a role in craniofacial development [45], and to coordinate with thyroid hormone and insulin-like growth factor (IGF) to affect bone mineral density [46,47].

Transcription factor enrichment analysis was performed, to identify TFs that may regulate expression of the DEGs, based on an over-representation of genes that contain TF-specific binding motifs. Two of the significantly enriched TFs, SUZ12 and EZH2, are members of the polycomb repressive complex 2, which operates as a regulator of transcriptional silencing through deposition of H3K27me3 histone marks, with important roles in transcriptional regulation of differentiation processes during development [48]. Of this pair, SUZ12 has not previously been associated with craniosynostosis, but there are several studies reporting associations between EZH2 and craniosynostosis. Deletion of *EZH2* in undifferentiated mesenchymal cells in developing mice caused multiple structural defects related to bone and skeletal patterning including craniosynostosis, limb shortening, and clinodactyly [49]. These defects were associated with changes to expression of HOX genes and may be attributable to early maturation of osteoblasts [49]. In our study, EZH2 was enriched as a regulator of DEGs associated with the lambdoid phenotype in our primary and male-stratified models, while this previous study found that *EZH2* deletion caused coronal and metopic craniosynostosis [49]. EZH2 has also been implicated in premature suture closure in craniosynostosis cases that are accompanied by a mutation in *TWIST1*, where EZH2 deposits fewer H3K27me3 silencing marks on osteogenic genes when *TWIST1* is deleted [50]. In our study, one of the significant downstream genes of EZH2 was *HOXB13*, which interestingly also acts as a TF, where it regulates the expression of *EZH2* itself, thus highlighting an interdependence of these two transcriptional regulators [51]. More work is needed to understand the relationship between these TFs and craniosynostosis etiology. 

Androgen receptor (AR) was significantly enriched as a transcriptional regulator for genes that were differentially expressed in sagittal craniosynostosis. This is noteworthy due to the 3x higher incidence of this phenotype in male patients compared to female patients in our study (Table 1). Dysregulation of androgen hormones has previously been implicated in both syndromic and non-syndromic cases of craniosynostosis [52] and the androgen receptor is abundantly expressed within the dura matter and calvarial bones of fetal mice [53]. Increased androgen hormones can also affect expression of TGF-beta, leading to increased osteoblast differentiation and premature suture fusion [54]. Multiple studies of suture closure in rabbits have indicated that treatment with the androgen-blocker flutamide can delay suture fusion and cause increased sutural separation [52,54]. In vitro treatment of fetal murine dural and osteoblast cells with androgen hormone 5-alpha dihydrotestosterone, has also been demonstrated to increase differentiation and proliferation, supporting androgen involvement in suture fusion [55]. 

This study should be interpreted with respect to its inherent limitations, including, that the sequencing was only performed on primary calvarial cells, consisting of mostly osteoblasts, whereas craniosynostosis likely involves gene expression changes to multiple cell types. Additionally, due to the rarity of craniosynostosis, some of the phenotypes, such as lambdoid, had limited sample size (N = 20). Another limitation is that the control group (N = 85) consisted of patients undergoing skull reconstruction surgeries for reasons unrelated to craniosynostosis, which could mean that there are baseline differences in calvaria bone gene expression between these controls and alternative non-pathological control sample populations, that did not have to undergo skull reconstruction surgery. Despite these limitations, this study had many strengths. One of the major strengths of this study is the sample size of 388 cases, spanning four phenotypes, which represents a large sample size of a relatively rare structural birth defect. This allowed us to individually analyze craniosynostosis phenotypes and perform a sex-stratified analysis for each phenotype. The use of RNA sequencing technology was also a strength, which aided in the identification of novel genes, pathways, and transcription factors related to craniosynostosis, that can be used to inform future studies.

## 4. Materials and Methods

### 4.1. Ethics Statement

This study was HIPAA compliant and independently approved by the Institutional Review Board (IRB) associated with each of the four clinical sites, including Seattle Children’s Hospital, Northwestern University in Chicago, Children’s Health Care of Atlanta, and St. Louis Children’s Hospital. Informed consent was obtained from parents or legal guardians of all participants with craniosynostosis. Waivers of consent were approved by Seattle Children’s Hospital, for anonymous control samples.

### 4.2. Study Participants

Three hundred and ninety-seven children, with computed tomography scans confirming diagnosis of single-suture craniosynostosis (SCC), were enrolled in the study at the time of treatment. Exclusion criteria included diagnosis of a major medical condition or presence of three or more minor extra-cranial malformations. Cases were also excluded based on the presence of causative mutations including *FGFR1*, *FGFR2*, *FGFR3*, *TWIST1*, *EFNB1*, and *MSX2.* Calvaria and blood samples were obtained from cases during surgery. Eighty-seven children were recruited as controls, with tissue collected during craniotomy procedures for reasons other than craniosynostosis (e.g., brain tumor, isolated hydrocephalus, or at the time of autopsy). Calvaria bone fragments from cases and controls were collected from otherwise discarded tissue. All cases were screened for known pathogenic variants in *FGFR1*, *FGFR2*, *FGFR3*, *TWIST1*, *EFNB1*, and *MSX2* and excluded from the analysis if carrying one of these pathogenic variants or a chromosomal rearrangement. Calvaria fragments were used to establish primary calvarial cell lines as described below. We also excluded samples with failed cell growth in culture. Only cases and controls with full covariate data were included in this analysis, resulting in a total study population of 473 participants, with 85 controls and 388 cases. 

### 4.3. Sample Processing

After collection, calvaria fragments were transported in Waymouth media (WM) (Sigma, St. Louis, MO, USA), supplemented with 2× antibiotic/antimycotic solution (GE Hyclone, Logan, UT, USA) and 10% fetal bovine serum (FBS) (GE Hyclone, Logan, UT, USA). To prepare the tissue for culture, tissue was rinsed in WM and surrounding soft tissue was removed. Calvaria fragments were cut with a sterile scalpel blade into 1–2 mm pieces. These small calvaria fragments were then cultured with two pieces per well in 12-well plates, in WM at 37 °C, 5% CO_2_, and 99% humidity. After reaching confluence, approximately 3–6 weeks after plating, cells were washed with PBS, trypsinized with 0.05% Trypsin-EDTA (GE Hyclone, Logan, UT, USA), and passaged into T75 flasks. Cells were grown to confluence in the T75 flask then cryogenically stored in liquid nitrogen, using freezing media containing 90% FBS and 10% dimethyl sulfoxide [25].

Once all samples were obtained and frozen, cells were thawed and cultured to sub-confluence in T25 flasks, where they were passaged at a density of 175,000 cells per 25 cm^2^. Once cells reached 75% confluence, they were trypsinized and washed in cold 1X PBS, followed by RNA isolation using the Roche High Pure miRNA Isolation Kit (Roche, Indianapolis, IN, USA), to isolate total RNA following the manufacturer’s protocol. The RNA Integrity Number (RIN) was assessed using the Agilent 2100 Bioanalyzer (Agilent Technologies, Santa Clara, CA, USA). Samples with RIN > 8.6 were used for RNA Sequencing [28]. 

### 4.4. RNA Sequencing

RNA was sequenced at Northwest Genomics Center, where next-generation sequencing libraries were prepared from 1.25 µg of total RNA in a high-throughput format, using the TruSeq Stranded mRNA kit (Illumina, San Diego, CA, USA). All the steps required for sequence library construction were automated and performed on a Sciclone NGSx Workstation (Perkin Elmer, Waltham, MA, USA). During library construction, rRNA was depleted by means of a poly-A enrichment, and first and second strand cDNA syntheses were performed. Each library was uniquely barcoded using Illumina adapters and amplified by PCR. After amplification and cleanup, library concentrations were quantified using the Quant-it dsDNA assay (Life Technologies, Carlsbad, CA, USA). Final libraries were normalized and pooled based on Agilent 2100 Bioanalyzer results (Agilent Technologies, Santa Clara, CA, USA) and size selected using a Pippin Prep (Sage Science, Beverly, MA, USA). Pooled libraries were diluted to a final concentration of 2–3 nM for sequencing on a HiSeq 4000, to a read depth of 30 million base pairs. Samples were multiplexed and sequenced on a HiSeq 4000. Lane-level sequencing reads were base quality checked using the FASTX-toolkit and FastQC, and aligned to hg19 with a reference transcriptome Ensembl v67, using TopHat2 suite [56] followed by matefixing, as described previously [28]. 

### 4.5. Differential Gene Expression Analysis

Genes were filtered, to remove non-protein coding genes and low expressing genes, using the EdgeR “filterbyExpr” function, with a cutoff of 20 cpm [57]. Linear models were constructed to identify differentially expressed genes (DEGs) for each phenotype, compared to the controls, using the limma-voom pipeline [58]. The primary model adjusted for patient sex, proband age, cell culture time, cohort, and sample city of origin. Surrogate variable analysis (SVA) was performed and included in the final model, to account for unknown confounding and cellular heterogeneity of samples [59]. Models stratified by patient sex were constructed using the same variables as the primary model, as well as model-specific surrogate variables. Adjustment for multiple comparisons was performed using the Benjamini–Hochberg method, and genes were considered significant when the false discovery rate (FDR) was < 0.05.

### 4.6. Pathway Enrichment Analysis

Pathway analysis was performed using the rotational self-contained gene set testing method, FRY, for each differential expression model described above [60]. FRY accounts for inter-gene correlations, by evaluating if the T statistic for a given gene is different than expected under the null hypothesis, and determines if an overall pathway is increased or decreased [60]. The Kyoto Encyclopedia of Genes and Genomes (KEGG) was used as the pathway database, and disease pathways were removed [61]. Pathways with FDR < 0.05 were considered significant. 

### 4.7. Transcription Factor Enrichment Analysis

Transcription factor enrichment analysis was performed on the lists of DEGs from the primary and sex-stratified phenotype models using Enrichr, with the ENCODE/ChEA consensus TF library [62,63,64]. TFs were considered significantly enriched when the FDR was < 0.05.

## 5. Conclusions

Overall, this study identified unique transcriptomic changes in primary cells derived from the calvaria of patients affected by four phenotypes of craniosynostosis. We also found sex-specific gene expression differences within the four individual phenotypes. This is particularly important, as metopic and sagittal phenotypes show higher occurrence in males, with coronal craniosynostosis occurring more frequently in females. Sixteen homeobox gene family members were associated with craniosynostosis phenotypes in our primary or male models. Differentially expressed genes for each phenotype were enriched for binding sites of TFs including AR, SUZ12, or EZH2 in primary and male models, highlighting a potential role for transcriptional regulation in eliciting altered gene expression. While many of the genes described in this paper have previous associations with craniosynostosis or osteoblast differentiation, many of these findings are novel, and will require additional experimental work to understand their role in craniosynostosis etiology. As the largest transcriptome-wide analysis of craniosynostosis to date, we anticipate that the results of this analysis will be utilized to generate new hypotheses on the molecular mechanisms underpinning craniosynostosis diagnoses.

## Figures and Tables

**Table 1:** Categorical covariate information for study participants. All participants included in this analysis (N = 473) had full covariate data.

	Control		Coronal		Sagittal		Metopic		Lambdoid		All	
	N	(%)	N	(%)	N	(%)	N	(%)	N	(%)	N	(%)
*Sex*												
Male	59	(18.55)	25	(7.86)	148	(46.54)	72	(22.64)	14	(4.40)	318	(100)
Female	26	(16.77)	55	(35.48)	48	(30.96)	20	(12.90)	6	(3.87)	155	(100)
*Sample Origin*												
Seattle, WA	55	(20.07)	45	(16.42)	113	(41.24)	50	(18.25)	11	(4.01)	274	(100)
St. Louis, MO	30	(25.42)	13	(11.02)	49	(41.53)	21	(17.80)	5	(4.24)	118	(100)
Chicago, IL	0	(0)	21	(38.18)	18	(32.73)	12	(21.82)	4	(7.27)	55	(100)
Atlanta, GA	0	(0)	1	(3.85)	16	(61.54)	9	(34.62)	0	(0)	26	(100)
*Cohort*												
1	48	(19.05)	49	(19.44)	96	(38.10)	48	(19.05)	11	(4.37)	252	(100)
2	37	(16.74)	31	(14.03)	100	(45.25)	44	(19.91)	9	(4.07)	221	(100)
**Total**	85	(17.97)	80	(16.91)	196	(41.44)	92	(19.45)	20	(4.23)	473	(100)

**Table 2:** Continuous covariate information for study participants. All participants included in this analysis (N = 473) had full covariate data.

		Control	Coronal	Sagittal	Metopic	Lambdoid	All
**Proband Age (months)**	Median	9	10	5.5	9	9	8
	Standard Error	3.17	0.75	0.40	0.46	0.68	0.66
	Range	0–120	1–59	1–36	1–25	2–15	0–120
**Culture Time (days)**	Median	44	42	34.5	37	49	39
	Standard Error	2.05	3.66	2.44	3.39	7.25	1.44
	Range	16–117	17–171	15–189	12–160	21–146	12–189

**Table 3:** Significant KEGG pathways (FDR < 0.05) for the combined-sex and sex-stratified phenotype models, identified through rotational gene set testing method FRY.

Phenotype	Model	Pathway	Direction	FDR
Coronal	Primary (N = 473)	Fatty acid degradation	Up	0.017
		Valine, leucine and isoleucine degradation	Up	0.017
		Butanoate metabolism	Up	0.019
Metopic	Male (N = 318)	Growth hormone synthesis, secretion and action	Up	0.028

## Data Availability

RNA sequencing data of craniosynostosis cases is available on dbGaP at accession number: phs002684.v1.p1. Code used to run the described analyses is available at https://github.com/SLapehn/Craniosynostosis_RNAseq.

## Supplementary Materials

The following supporting information can be downloaded at: https://www.mdpi.com/article/10.3390/ijms24065353/s1.
