# Supplementary material for: Transcriptomic Signatures of Single-Suture Craniosynostosis Phenotypes
Source: Int J Mol Sci. 2023 Mar 10;24(6):5353. doi: 10.3390/ijms24065353 (PMC10049207; doi:10.3390/ijms24065353)
Supplement: Supplementary file 1 [file ijms-24-05353-s001.zip › ijms-2257463-supplementary Figures S1 and S2.pdf]

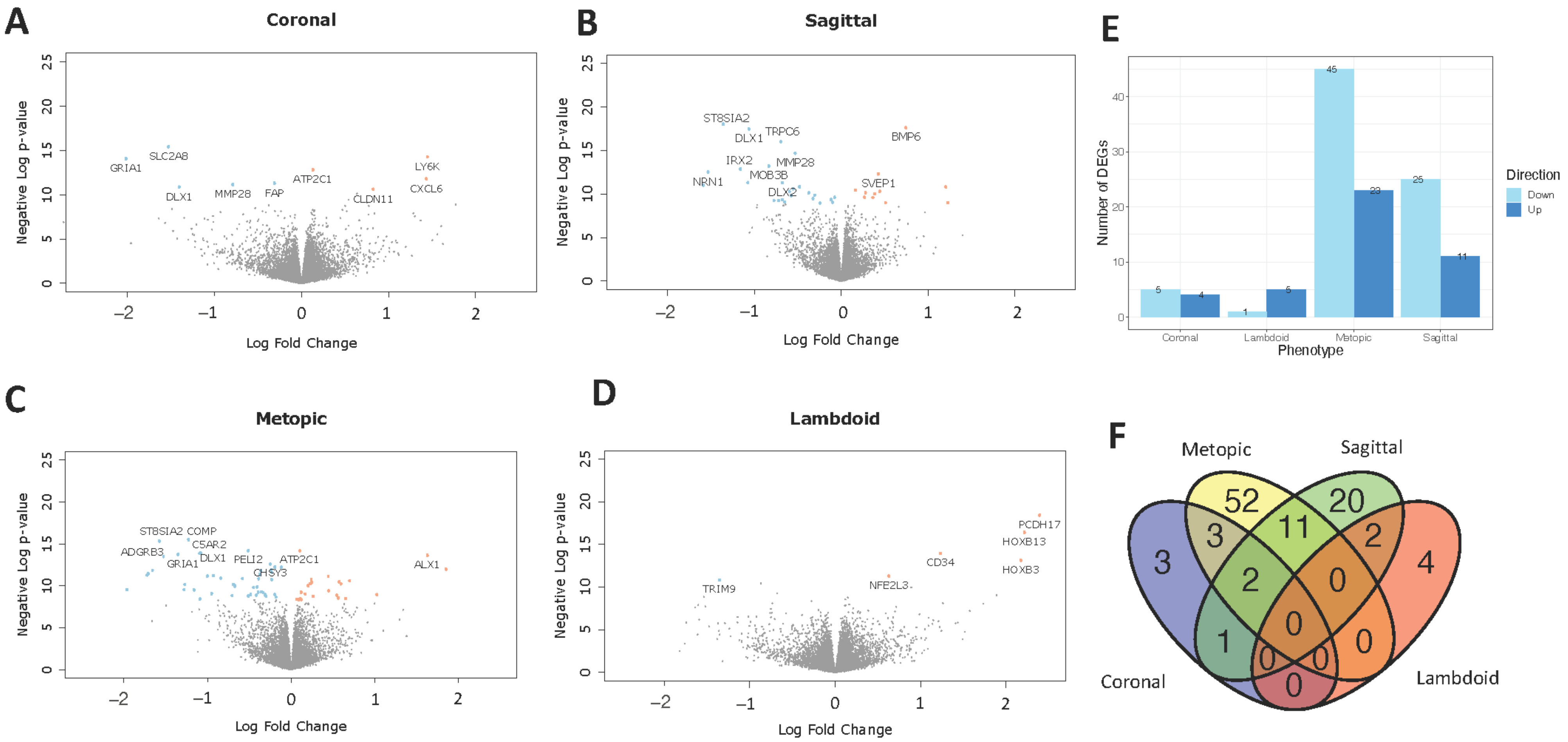

Supplemental Figure S1. Differentially expressed genes (DEGs) (FDR<0.05) for the male-stratified phenotype model. DEGs are represented blue (decreased) or orange (increased) points. The top 10 DEGs based on log fold change are labeled by gene ID. A) Coronal phenotype B) Sagittal phenotype C) Metopic phenotype D) Lambdoid phenotype E) Number and direction of DEGs for each phenotype F) Overlap of DEGs across phenotypes.

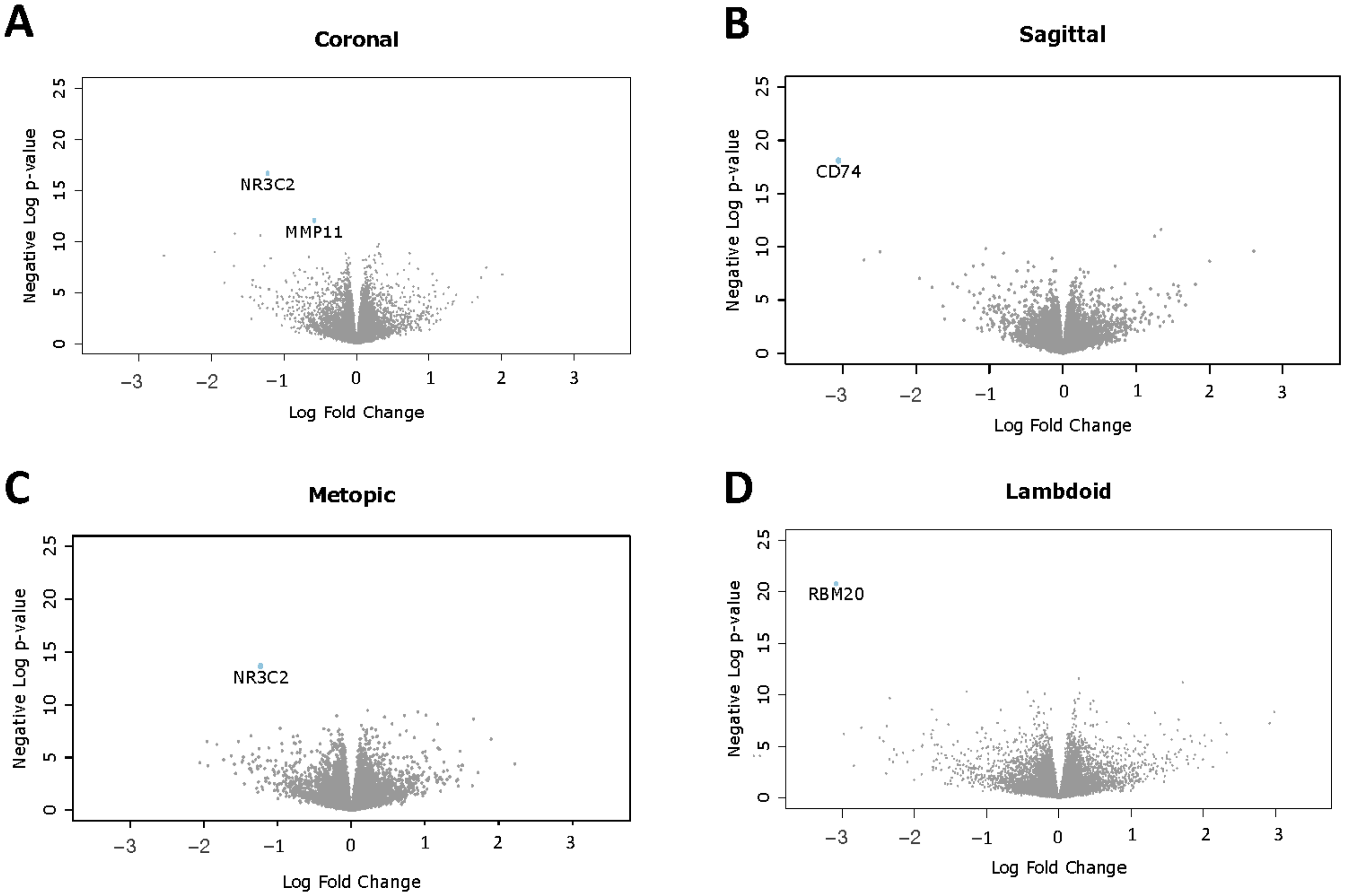

Supplemental Figure S2. Differentially expressed genes (DEGs) (FDR<0.05) for the female-stratified phenotype model. DEGs are represented blue (decreased) or orange (increased) points. A) Coronal phenotype DEGs B) Sagittal phenotype DEGs C) Metopic phenotype DEGs D) Lambdoid phenotype DEGs.
